# In-vitro toxicity study of poly(alkylphenol) as vulcanizing agent

**DOI:** 10.1186/s40824-016-0082-2

**Published:** 2016-11-14

**Authors:** Joon Woo Chon, Ji Yun Lee, Yoon Ju Song, Jin Hwan Kim, Dong June Chung

**Affiliations:** 1 Department of Polymer Science and Engineering, Sungkyunkwan University, Suwon, 16419 Korea; 2 M&B GreenUs Co, Ltd, #1016, 278, Beotkkot-ro, Geumcheon-gu, Seoul 08511 Korea

**Keywords:** Elastic rubber, Medical application, Alkyl phenol vulcanizing agent, Cytotoxicity

## Abstract

In this study, cytotoxicity of various novel poly(alkylpehnol) derivatives which, one of constituent for vulcanizing agent, could be adjusted in medical elastic rubber applications were investigated under various conditions of cytotoxicity test.

By MTT-assay which according to ISO 10993–5 regulation, we could figure out cell viability of mouse fibroblast in various sample conditions. Furthermore, by Live & Dead Cell assay, we could get colorimetric cell viability via fluorescence images.

## Background

Elastic rubbers are used numerous applications in modern life and in many types of clinical equipments [1, 2]. The use of elastic rubber for medical devices was begun when the rubber industry started especially after the discovery of vulcanized natural rubber. The elasticity and flexibility make it good approach to medical application [3]. Elastic rubber was first used in medical devices in the sealing cab of disposable medical syringe and therapeutic injection ampoules. The vulcanizing agent is one of the most important constituent of rubber and establishes its properties during mixing and molding. After vulcanization, its mechanical properties (elasticity and flexibility) are enhanced. In addition to these properties, biocompatibility and non-toxicity are especially important for clinical applications of medical rubber. However vulcanizing agents that contain nitrogen frequently produced nitrosamine as a decomposition product when the rubber waste was incinerated; this is undesirable because nitrosamine is toxic to humans [4]. Furthermore, nitrosamine is carciogenic [5, 6]. Thus, the development of nitrogen-free vulcanizing agent is one of the most important challenges for preventing potential toxicological issues.

To overcome this problem, poly(alkylphenol) disulfide reagents are believed to be promising nontoxic vulcanizing agent. Poly(alkylphenol) sulfide which derived from various configuration of alkyl group with sulfur content has potential double sulfur bridges so that many great deal on vulcanizing agent [7].

Furthermore, one of important benefit by using poly(alkylphenol) sulfide as vulcanizing agent to adjust medical device is that these chemical compounds are not only nitrosamine free but also better heat resistance, dynamic fatigue properties, and enhanced adhesion [8].

## Main text

### Materials and methods

**Fig. 1:**
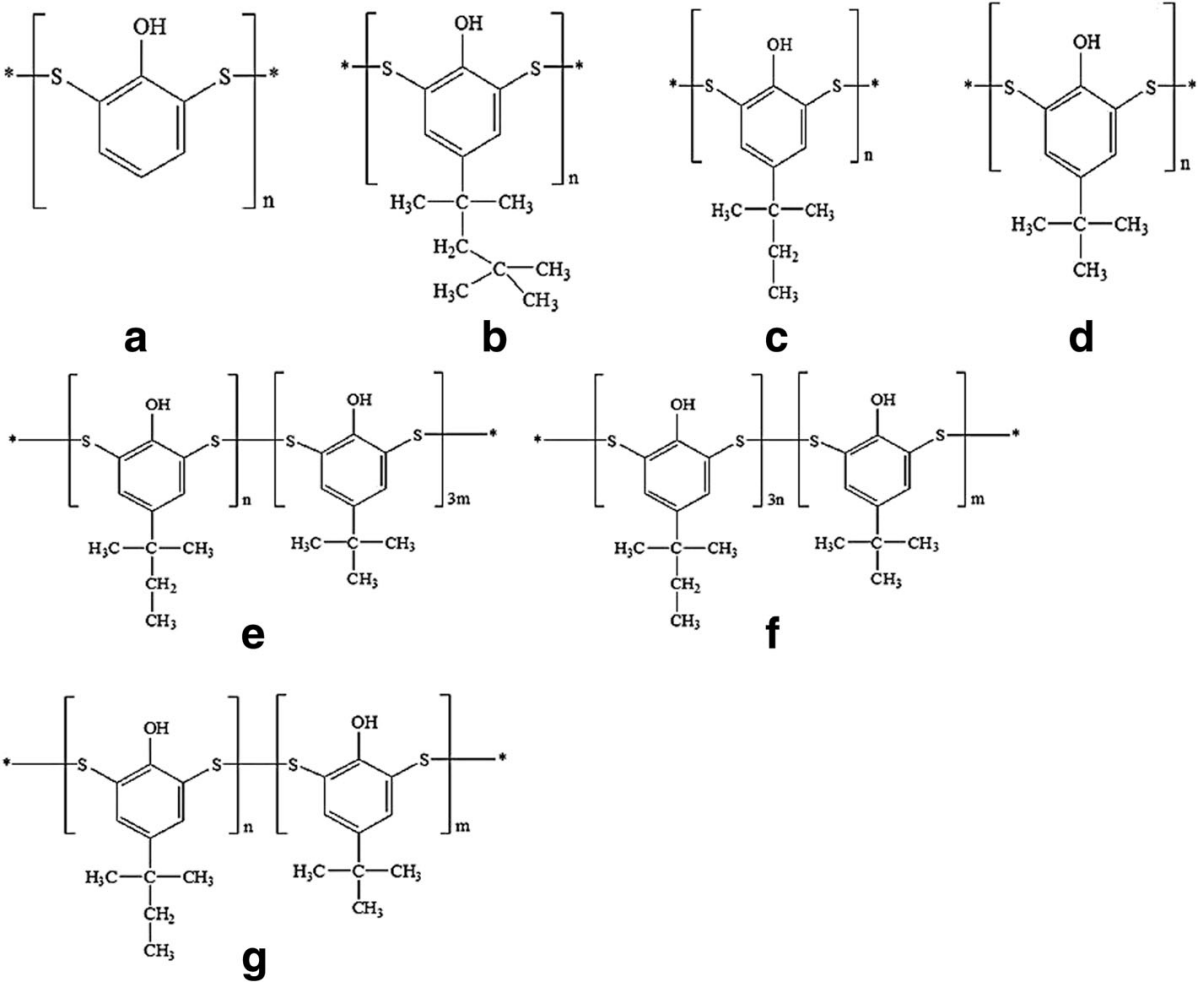
Chemical structures of synthesized disulfide samples for vulcanizing agent. **a** (+) Control, **b** P 1, **c** P 2, **d** P 3, **e** CP-1; Copolymer of P 2 and P 3 (1:3, monomer mol ratio in feed), **f** CP- 2; Copolymer of P 2 and P 3 (3:1, monomer mol ratio in feed), **g** CP-3; Copolymer of P 2 and P 3 (1:1, monomer mol ratio in feed)

We investigated the cytotoxicity of various poly(alkylphenol) disulfide vulcanizing agents, with the aim of using these agents for clinical application. Novel poly(alkylphenol) derivatives were synthesized and kindly supplied by M&B GreenUs Co. (Seoul, Korea) (Fig. 1 and Table 1). For the synthesis of polyphenol, P1, P2 and P3, the appropriate monomers (phenol, *p*-tert-octylphenol, *p*-(1,1-dimethylethyl) phenol, and *p*-tert-butylphenol, respectively) were reacted with S_2_Cl_2_ in toluene at 150 ~ 180 °C/4 ~ 6 h to obtain macromolecules by forming sulfuric bridge between the monomers. To control the temperature of the exothermic reaction, S_2_Cl_2_ solution was slowly added by dropwise using a funnel. Gaseous HCl, generated during the formation of S-S bonds, was removed using an HCl trap under a nitrogen stream. The resultant polymers were separated by distillation at 200 °C for 0.5 ~ 1 h. For the synthesis of novel copolymer type vulcanizing agent (CP-1, CP-2, and CP-3), two monomers (*p*-(1,1-dimethylethyl) phenol and *p*-tert-butylphenol) were copolymerized with S_2_Cl_2_, under the same reaction conditions mentioned above, in various molar feeding ratios (Table 1). The molecular weight(Mw) of synthesized homopolymer and copolymer was measured GPC(eluent; THF, eluting rate; 0.8 ml/min, column; Styragel Guard column, Styragel HR(High Resolution), Styragel 4E and Styragel 5E by Waters Co, Ltd., Milford, MA, USA). The dermal LD_50_ values in rabbits of these two monomers are lower than those of pure phenol and therefore *p*-tert-octylphenol [9, 10], *p*-(1,1-dimethylethyl)phenol and *p*-tert-butylphenol were selected as nontoxic vulcanizing agent for copolymer synthesis and their cytotoxicity was compared to that of control samples. Pure phenol, PBS (phosphate buffered solution), and synthesized polyphenol were used as control.

**Table 1:** Various formulation ratios of samples

Sample	Constituent	Solvent for samples	Sample concentrations in DMSO (mol %)	Dilution ratio with PBS (wt %)	Mw measured by GPC
PBS	Phosphate Buffered Solution	DMSO	-	-	-
(−) control	Pure phenol (Sigma-Aldrich Co., Ltd., St. Louis, USA)	DMSO	1 %, 5 %, 10 %	1 %, 5 %, 10 %	-
(+) control	Poly phenol disulfide (GreenUS M&B Co., Seoul, Korea)	DMSO	1 %, 5 % 10 %	1 %, 5 %, 10 %	802
P1	Poly *p*-octylphenol disulfide (GreenUS M&B Co., Seoul, Korea)	DMSO	1 %, 5 %, 10 %	1 %, 5 %, 10 %	2,027
P2	Poly *p*-(1,1 dimethyl ethyl) phenol disulfide (GreenUS M&B Co., Seoul, Korea)	DMSO	1 %, 5 %, 10 %	1 %, 5 %, 10 %	2,116
P3	Pol *p*-tert-butylphenol disul fi de (GreenUS M&B Co., Seoul, Korea)	DMSO	1 %, 5 %, 10 %	1 %, 5 %, 10 %	2,365
CP-1	Copolymer of P2 and P3 (1:3, in monomer feeding ratio) (GreenUS M&B Co., Seoul, Korea)	DMSO	1 %, 5 %, 10 %	1 %, 5 %, 10 %	2,772
CP-2	Copolymer of P2 and P3 (3:1, in monomer feeding ratio) (GreenUS M&B Co., Seoul, Korea)	DMSO	1 %, 5 %, 10 %	1 %, 5 %, 10 %	2,728
CP-3	Copolymer of P2 and P3 (1:1, in monomer feeding ratio) (GreenUS M&B Co., Seoul, Korea)	DMSO	1 %, 5 %, 10 %	1 %, 5 %, 10 %	2,632

To compare the cytotoxic effects of the vulcanizing agents like as synthesized polymeric vulcanizing agents, phenol((−) control), and polyphenol disulfide((+) control), 9 samples were dissolved in DMSO at 1 mol %, 5 mol %, and 10 mol % for 1 h at room temperature. These sample solutions (PBS, (+) control, (−) control and the 6 samples) were then diluted in PBS to 1 wt %, 5 wt %, and 10 wt % for cytotoxicity assays using fibroblasts. The MTT assay and the Live & Dead Cell assay were conducted to evaluate the cytotoxicity of each compound. These assays work by analyzing cell viability [11, 12]. Commercial kits were used for the two assays (Sigma-Aldrich Co., Ltd., St. Louis, MO, USA). All experiments were carried out 5 times. Briefly, L-929 mouse fibroblasts (obtained from the Korean Cell Line Bank Co., Korea) were seeded (1 × 10^5^ cell/mL) into each well of a tissue culture polystyrene dish (TCPS). The cell were incubated at 36.5 °C in a 5 % CO_2_ with 10 % FBS (fatal bovine serum) and 1 % penicillin-streptomycin. For the assay, the 9 solutions described above were added to the TCPS wells and incubated with cells for 2 days. The MTT assay was performed according to the ISO 10993-5 guidence [13]. Briefly, DMEM and MTT were added to each well and the mixture was incubated for 4 h under the same conditions. After this incubation, the supernatant was removed from each well by aspiration. Next, DMSO and glycine buffer were sequentially added to each well. Finally, the UV–Vis absorbance of each well was measured at 570 nm using a SpectraMax M5 plate reader (Molecular Devices, Co., Seoul, Korea). For the Live & Dead Cell assay, samples were prepared as for the MTT assay. Next, calcein-AM and propidium iodide (PI) were added to each well and incubate for 15 min at 36.5 °C in 5 % CO_2_ atmosphere. Fluorescence images were captured and analyzed using a Nikon Eclipse Ti microscope (Nikon Instruments Inc., Tokyo, Japan). Furthermore, ALP assay was carried out with Alkaline Phosphatase Assay Kit (AnaSpec Co., Inc., Fremont, CA, USA) to determine changes in activity of phosphatase which derived from cell membrane [14, 15]. The biological sample treating was more harsh than MTT assay and Live & Dead Cell assay. Concentration range of every samples for ALP assay were 10 mol % to 40 mol %. Each activity of phosphatase were measured at 405 nm using a SpectraMax M5 plate reader (Molecular Devices, Co., Seoul, Korea).

### Results and discussion

**Fig. 2:**
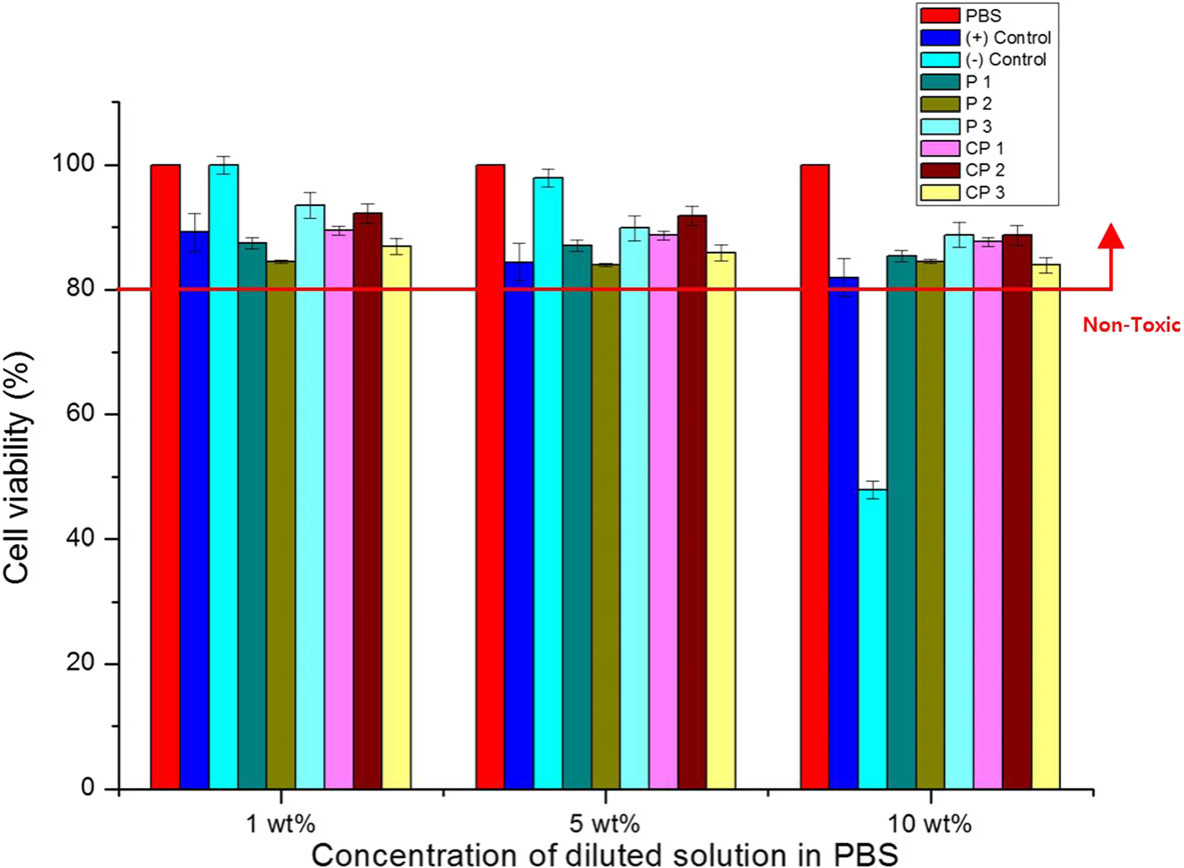
Cell viability of 1 mol % of disulfide sample solutions

The MTT assay is one of the most powerful method for analyzing cell viability and is based on a colorimetric assay that measures the metabolic activity in living cells. In this asssy, MTT tetrazolium (yellow) is reduced to MTT formazan, which becomes purple after mitochondrial reduction in living cells. The UV–Vis absorbance change at 570 nm is directly proportional to the amount of MTT formazan reduction. As shown in Fig. 2, treatment with the 1 mol % concentration of the disulfide sample solutions (6 kinds) and of the (+) control sample (each of which was diluted by PBS to 1, 5, and 10 wt%) resulted in over 80 % viability. Strikingly, this viability was maintained even up to the 10 wt % dilution. These results indicate that all samples (with the exception of the (−) control at 10 wt%) can be classified as nontoxic according to the ISO guidelines. In contrast, the viability of cells treated with pure phenol, which was used as negative control, decreased rapidly to less than 50 % for the 10 wt% dilution. These results demonstrate the non-cytotoxicity of the 6 synthesized compounds and the (+) control(polyphenol), in contrast to the (−) control (pure phenol). In particular, cells treated with the copolymer samples showed enhanced viability compared to cells treated with the (+) control, even up though the 10 wt% dilution.

**Fig. 3:**
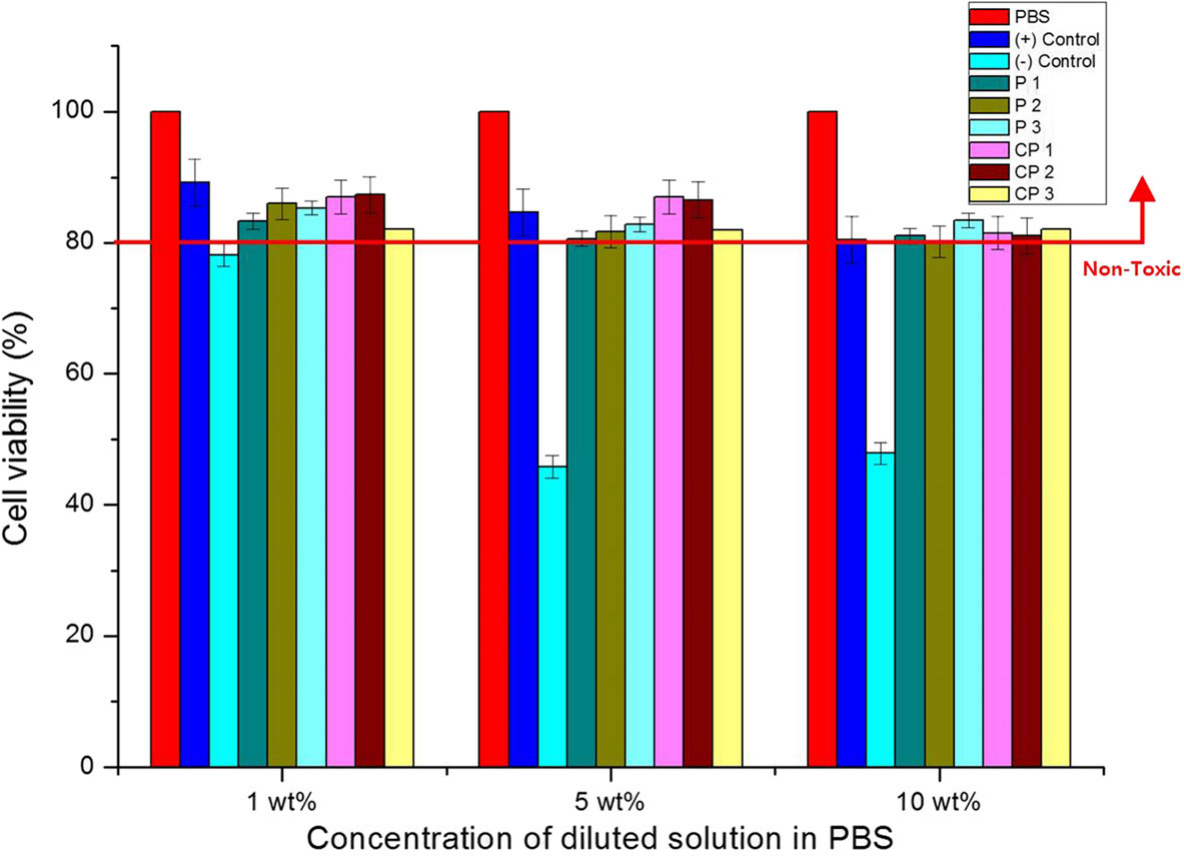
Cell viability of 10 mol % of disulfide sample solutions

We next tested the cytotoxicity of 10 mol % concentrations of the disulfide sample solutions, a relatively high concentration considering the typical concentrations of vulcanizing agents in rubber processing. As shown in Fig. 3, the overall viabilities of cells treated with the various solutions (each of which was diluted to 1 wt%, 5 wt%, and 10 wt% in PBS) at 10 mol % were decreased compared with the corresponding viabilities of the cells treated with the 1 mol % solutions. However, cell viability always remained above 80 % comparing to that of control at the 10 wt% dilutions, with the exception of cells treated with the polymeric vulcanizing agents, which were close to 80 % viable after treatment with the 5 and 10 wt% dilutions. This means that the poly(alkylphenol) samples were toxic at high concentrations (10 mol %). However, the viability of cells treated with pure phenol showed a drastic decrease. Furthermore, compared with cells treated with 10 mol % of a disulfide sample solution, cells treated with pure phenol (even diluted phenol) showed much lower viability.

We also performed a colorimetric cell viability assay as a complementary technique. To this end, we used the Live & Dead Cell assay. Briefly, 1 mol % disulfide solutions were prepared by diluting the 10 wt % solutions with PBS. The treated samples were observed by fluorescence microscopy. The two probes used in this assay have different fluorescence colors based on whether they recognize live or dead cells. Live cells are distinguished by green fluorescence, which results from esterase-mediated hydrolysis of the acetoxymethyl ester bond in calcein AM. On the other hand, the ethidium homodimer binds to DNA in dead cells and produces red fluorescence.

**Fig. 4:**
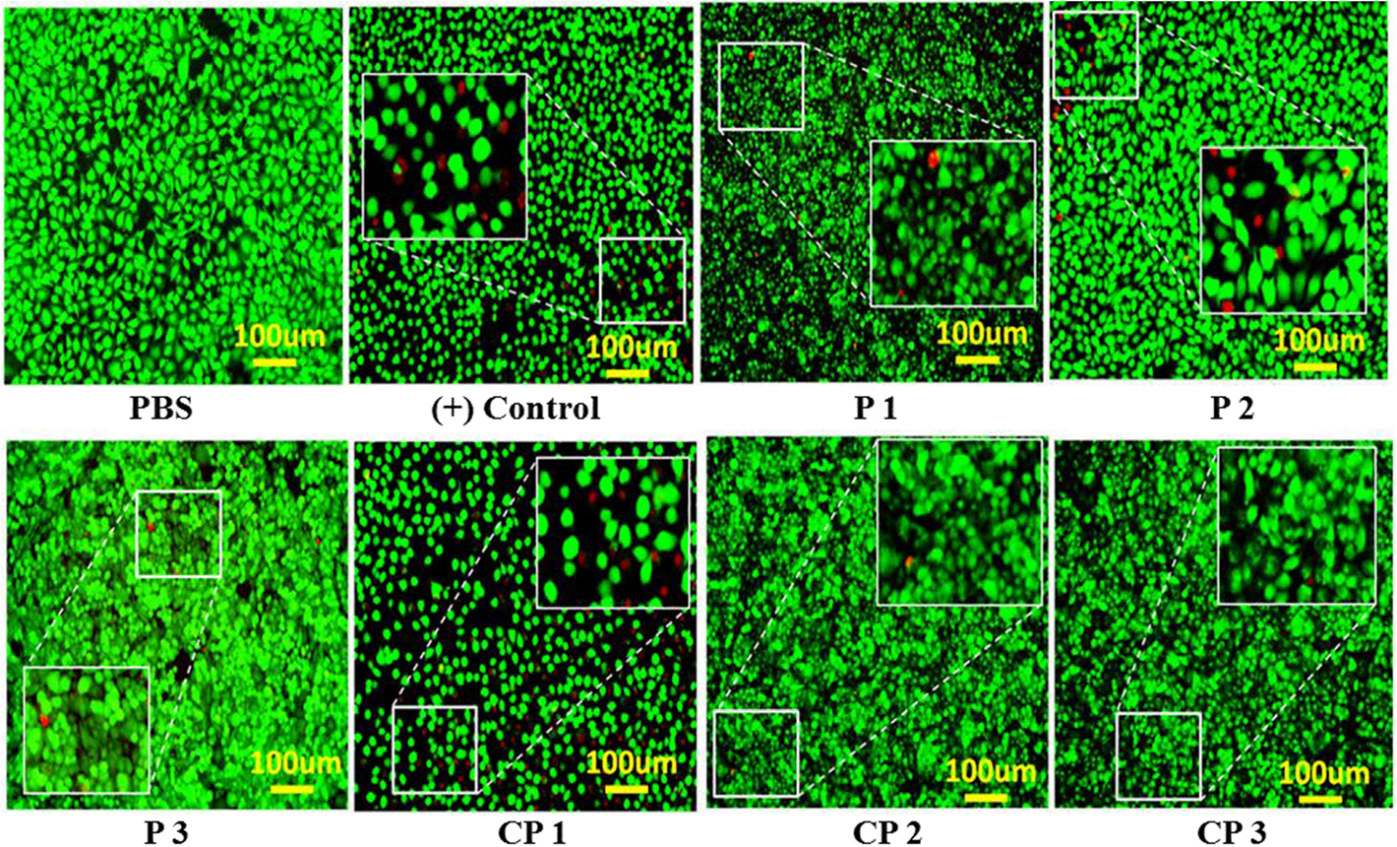
Image of Cell viability of 1 mol % of disulfide solution via Live & Dead Cell-assay (Diluted by PBS in 10 wt %). Dead cell (*Red Spot*) was emphasized with *square mark*

As shown in Fig. 4, red cells were observed in many of the TCPS wells, but most of the cells in the TCPS were green after each of the predetermined culture times. This finding indicates that most of the cells remained alive after treatment with the 1 mol % solutions (which had been generated from the 10 wt % solutions by dilution with PBS). Comparison of the proportions of dead cells (red fluorescence) to live cells (green fluorescence) in the images of the homopolymer (P1 ~ P3) and copolymer (CP-1 ~ CP-3)-treated wells revealed that the copolymers were much less cytotoxic than the homopolymers. These results are consistant with the MTT assay results (Fig. 2), which indicate that the copolymer are all nontoxic.

**Fig. 5:**
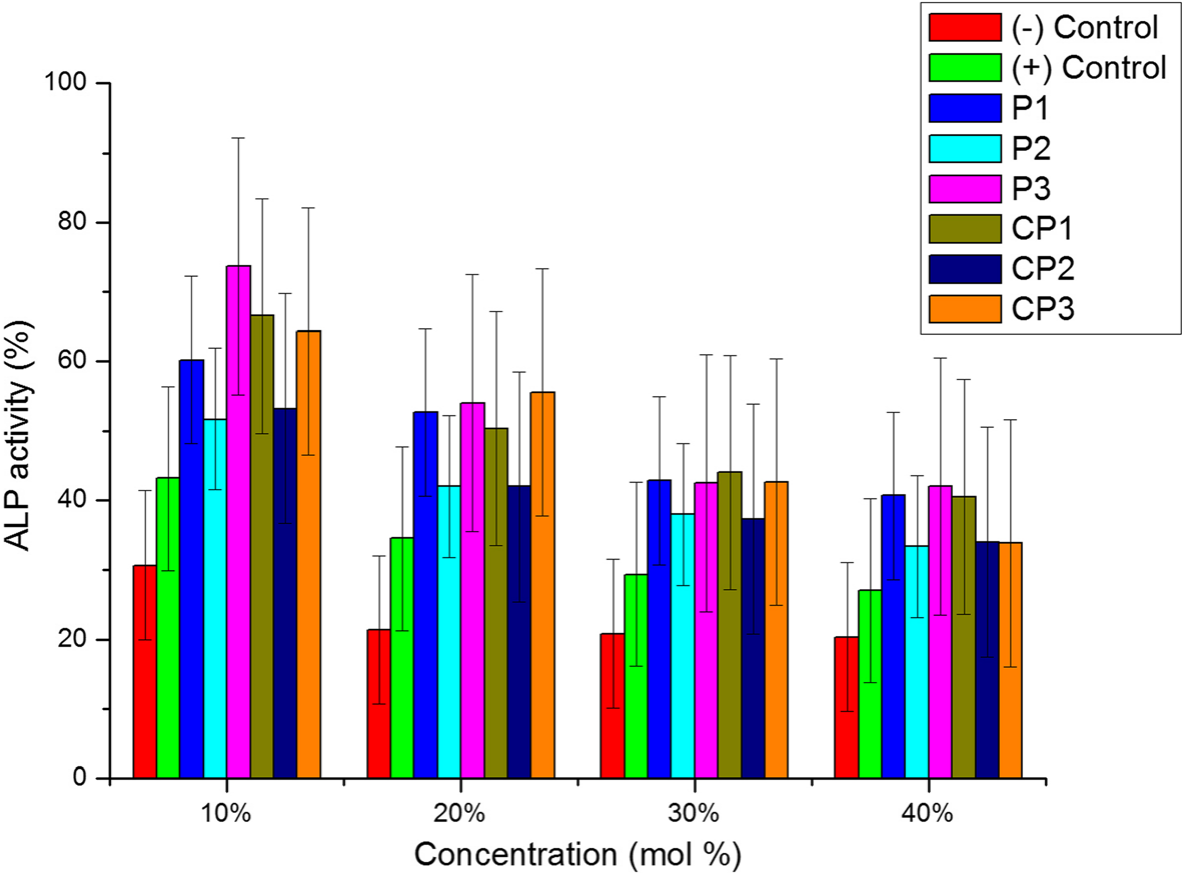
ALP activity of various mol % of each samples

From Fig. 5, ALP activity of every samples were marked higher level than the (−) control (Pure phenol) at 10 mol % of concentration in DMSO. Even concentration increment till 40 mol %, ALP activity of homopolymers and copolymers still marked higher level than the (−) control. These results indicated that cytotoxicity of each samples showed generally similar tendency with MTT-assay and Live & Dead Cell assay.

## Conclusion

In this study, we used the MTT assay and the Live & Dead Cell assay to test the cytotoxicity of various polymeric disulfide samples that could potentially be used as vulcanizing agent in medical elastic rubber. Every synthesized sample was classified as nontoxic according to the ISO standards. Fluorescence microscopy revealed many live cells on the TCPS, even after treatment with the disulfide sample solutions. Furthermore, each sample was less cytotoxic than pure phenol, and yielded similar viability compared with the (+) control under the same diluting conditions. From ALP assay, every sample showed higher activity level than both control even concentration increased. Further work will include to in-vivo experiments of toxicity of our synthesized disulfide samples. To this end, skin sensitization test and inflammation analysis will be carried out in animal modles to determine the biological response to our disulfide samples.

## Abbreviations

CP-1: Copolymer of P2 and P3 (1:3, in monomer feeding ratio)
CP-2: Copolymer of P2 and P3 (3:1, in monomer feeding ratio)
CP-3: Copolymer of P2 and P3 (1:1, in monomer feeding ratio)
P1: Poly *p*-octylphenol disulfide
P2: Poly *p*-(1,1dimethylethyl)phenol disulfide
P3: Poly *p*-tert-butylphenol disulfide
